# Extrusion-Induced Gelation and Network Formation in Meat Analogs Produced from Mung Bean Protein

**DOI:** 10.3390/gels12020102

**Published:** 2026-01-26

**Authors:** Yu Zhang, Nam-Ki Hwang, Gi-Hyung Ryu, Bon-Jae Gu

**Affiliations:** 1 Department of Food and Quality Engineering, Nanning University, Nanning 530200, China; 2 Department of Food Science and Technology, Food and Feed Extrusion Research Center, Kongju National University, Yesan 32439, Republic of Korea

**Keywords:** extrusion-induced gelation, protein network formation, gel-like structure, structure-property relationship, low-moisture extrusion, plant-based meat analogs

## Abstract

Extrusion processing can induce gel-like network formation in plant proteins, enabling the advancement of structured meat alternatives with tailored textural properties. In this study, extrusion-induced gelation behavior of isolated mung bean protein (IMBP) was systematically investigated during the manufacture of low-moisture meat analogs (LMMA). The effects of key processing variables, rotational speed of the screw, moisture level, and processing temperature on gel network development, hydration behavior, and textural responses were evaluated using response surface methodology as an analytical framework. Increasing moisture content promoted protein hydration and facilitated the formation of continuous gel-like interactions, resulting in enhanced pore development and water-holding capacity. Variations in screw speed and processing temperature further modulated the extent of protein denaturation and network consolidation, influencing nitrogen solubility and mechanical properties. While the integrity index remained relatively insensitive to processing conditions, structural and functional responses exhibited clear dependencies on extrusion-induced gelation dynamics. The extrusion conditions of 39% moisture, 216 rpm, and 159 °C promoted the development of a well-defined protein network, leading to improved functional properties. These findings provide mechanistic insight into extrusion-driven gelation of IMBP and highlight its potential as a protein matrix for gel-based meat analog applications.

## 1. Introduction

Protein-based meat analogs have received considerable attention as structured food systems capable of addressing sustainability, health, and resource-efficiency challenges associated with conventional meat production. Growing demand for alternative protein sources has been driven by concerns regarding land and water consumption, environmental burden, and dietary health, leading to increased interest in plant-derived protein matrices that can deliver meat-like texture and functionality [1,2,3,4]. Among various plant protein sources, mung bean (*Vigna radiata* L.) has emerged as a promising raw material due to its favorable nutritional profile, cholesterol-free composition, and functional versatility. Mung bean proteins exhibit notable gelling and emulsifying abilities, enabling the formation of structured matrices under appropriate thermal and mechanical treatments [5,6,7,8]. In addition, the long cultivation history, stable yield, and well-established processing infrastructure of mung bean further support its applicability in structured protein-based foods. Previous studies have demonstrated that texturized mung bean protein can achieve desirable physical and textural attributes, highlighting its potential as a functional protein matrix in meat analog formulations [9].

Extrusion processing represents one of the most effective approaches for inducing structural transformation in plant proteins. Through the combined engagement of thermal energy, pressure, and mechanical shear, extrusion promotes protein unfolding, aggregation, and rearrangement, resulting in the development of organized, gel-like or fibrous structures upon cooling [10]. The extent and nature of this structural development are strongly influenced by processing variables, including rotational speed of the screw, moisture level, and processing temperature, which collectively regulate protein mobility, denaturation kinetics, and network formation [11,12]. Numerous studies have reported that modulation of these parameters governs expansion behavior, textural strength, and hydration properties in extruded protein systems [13,14,15,16]. While high-moisture extrusion has been extensively explored for generating anisotropic fibrous networks, low-moisture extrusion constitutes a distinct processing regime in which limited water availability alters protein–protein interactions and gelation pathways. In such systems, subtle variations in processing conditions can lead to pronounced differences in network continuity, pore structure, and functional performance. Despite increasing interest in mung bean protein for manufacturing meat alternatives, systematic investigations focusing on extrusion-induced gelation behavior of mung bean protein under low-moisture conditions remain limited [6,17,18].

Therefore, the goal of this study was to elucidate the influences of key extrusion parameters, moisture level, screw speed, and processing temperature, on gel network development and structure–property relationships in meat analogs formulated with mung bean protein. By applying the response surface modeling approach as an analytical framework, this work aims to provide mechanistic insight into extrusion-induced gelation and to establish processing–structure–function relationships relevant to gel-based plant protein systems.

## 2. Results and Discussion

### 2.1. Cross-Sectional Morphology

**Figure 1:**
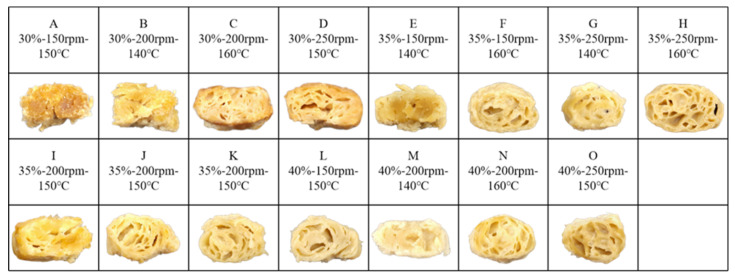
Cross-sectional images of meat analogs produced across a range of extrusion parameter settings.

Figure 1 illustrates the cross-sectional morphology of meat analogs from mung bean protein isolate manufactured under various extrusion conditions. Distinct variations in pore size and pore density were observed as functions of rotational speed of the screw, moisture level, and processing temperature. Overall, increases in these processing variables promoted more pronounced porous structures within the extrudates. An increase in moisture content resulted in larger and more numerous pores in the IMBP-based LMMAs. Under elevated temperature and pressure conditions inside the extruder, water present in the feed material undergoes rapid phase transition, generating steam that expands the molten matrix during extrusion. As the extrudate exits the die and cools, these steam bubbles become entrapped, forming pores within the solidified structure [19]. Similar trends have been reported for protein-enriched peanut-based meat analogs, where higher feed moisture led to enhanced expansion behavior and increased pore development [14]. Screw speed also played a critical role in determining pore characteristics. Higher screw speeds intensified mechanical shear and mixing efficiency, leading to more homogeneous temperature distribution and increased incorporation of air bubbles into the molten protein matrix. As a result, the extrudates exhibited a higher number of uniformly distributed pores. Barrel temperature exerted a pronounced influence on expansion behavior and internal structure formation. Elevated temperatures increased the thermal energy of water molecules, reduced melt viscosity, and facilitated bubble growth during extrusion [20].

Consequently, higher barrel temperatures promoted greater expansion and porosity in the low-moisture meat analogs. Consistent observations were reported for cereal–legume extrusion systems, where increased barrel temperature significantly improved expansion characteristics [21]. Furthermore, higher thermal input accelerated water evaporation, generating additional steam and contributing to the formation of larger pores within the extrudate matrix.

### 2.2. Water Retention Behavior

Water retention properties of the meat analogs varied markedly depending on the applied extrusion conditions, as summarized in Table 1. Overall, WHC increased with higher rotational speed of the screw, moisture level, and processing temperature, showing trends consistent with the structural features observed in the appearance analysis. Samples characterized by larger pore sizes and higher pore density generally exhibited enhanced WHC, indicating a close relationship between internal porous structure and water retention ability [17,22]. During extrusion, water acts as both a plasticizing and structuring medium, participating in protein hydration, dispersion, and gel network formation. Adequate moisture facilitates protein unfolding and intermolecular interactions, promoting gelation while simultaneously occupying void spaces within the developing protein network. As a result, higher moisture content favors the formation of a more continuous and homogeneous gel-like matrix, which enhances the ability of LMMAs to immobilize water within their structure. Screw speed also contributed positively to WHC. Increased screw speed intensified mechanical shear and expansion behavior, generating a greater number of fine pores and voids within the extrudate.

These microstructural features increased the effective surface area and provided additional sites for water entrapment, leading to improved water retention. Barrel temperature exerted a pronounced influence on WHC through its effect on protein denaturation and network consolidation. Elevated temperatures promoted unfolding protein and cross-linking, developing the formation of a stronger three-dimensional gel interaction capable of retaining water more effectively. Similar behavior has been reported for extruded soy-based systems, where high barrel temperature enhanced WHC despite relatively low feed moisture [23]. Collectively, these results demonstrate that WHC in IMBP-based LMMAs is governed by extrusion-induced gel network development and the associated microstructural characteristics.

### 2.3. Integrity Index and Nitrogen Solubility Index

Integrity index and nitrogen solubility index (NSI) were assessed for assessing the structural stability and molecular state of proteins in meat analogs following extrusion. While the integrity index reflects the resistance of the extruded matrix to mechanical disruption during rehydration, NSI provides insight into the extent of protein denaturation and aggregation induced by thermal and mechanical processing. As shown in Table 1, integrity index of IMBP-based LMMAs was not significantly influenced by extrusion variables, including rotational speed of the screw, moisture level, and processing temperature (*p* > 0.05). In particular, variations in barrel temperatures resulted in negligible differences in integrity index values among comparable samples (e.g., samples 5 and 6, 10 and 11, and 13 and 14). These results indicate that within the investigated processing window, the overall cohesion of the protein network was sufficiently robust to withstand rehydration and mechanical dispersion, resulting in limited solids loss [7,16]. In contrast, NSI exhibited greater sensitivity to extrusion conditions. As summarized in Table 2, NSI generally decreased with increasing mechanical and thermal severity, particularly with higher screw speed and reduced moisture availability. This trend suggests enhanced protein denaturation and aggregation during extrusion, leading to a shift from soluble protein fractions toward insoluble, network-forming aggregates [6,17].

Moisture content played a critical role in modulating this behavior; adequate hydration facilitated protein unfolding and molecular mobility, whereas restricted water availability intensified protein–protein interactions, promoting aggregation and reduced nitrogen solubility. Notably, the absence of significant changes in integrity index despite variations in NSI suggests that extrusion-induced protein aggregation primarily influenced molecular solubility rather than macroscopic network cohesion. In other words, while NSI reflected conformational and intermolecular changes at the protein level, the three-dimensional gel-like network formed during extrusion remained structurally stable across the tested processing conditions. This decoupling of protein solubility and structural integrity highlights the formation of a consolidated protein network capable of maintaining mechanical robustness even as soluble protein fractions decreased. Taken together, the combined analysis of integrity index and NSI indicates that extrusion-induced aggregation of mung bean protein contributes to the development of a stable, insoluble protein network without compromising structural integrity. These findings support the role of controlled protein denaturation and aggregation as key mechanisms governing structure–property relationships in IMBP-based meat alternatives.

### 2.4. Mechanical and Textural Properties

The textural characteristics of extruded products are closely associated with sensory perception and are largely governed by the internal structural organization of the material [24,25]. Table 2 presents the effect of extrusion processing variables on texture profile parameters and cutting strength of meat alternatives. At relatively low screw speed and barrel temperature (e.g., samples 1 and 12, and samples 2 and 13), increasing moisture level led to significant increases (*p* < 0.05) in chewiness, springiness, cohesiveness, and cutting strength. This behavior can be attributed to enhanced hydration and plasticization of the protein matrix, which facilitated network development and improved elastic recovery. Similar trends have been reported for cereal- and millet-based extruded products, where higher moisture content increased hardness and, consequently, chewiness [26,27]. In contrast, under conditions of high screw speed (samples 4 and 15) or elevated barrel temperature (samples 3 and 14), increasing moisture level caused a significant reduction (*p* < 0.05) in chewiness, while other textural parameters exhibited no significant changes (*p* > 0.05). This suggests that excessive moisture, when combined with high mechanical or thermal input, may weaken network resistance to deformation by promoting over-plasticization of the protein matrix. Screw speed generally exhibited a positive relationship with TPA parameters and cutting strength, reflecting the role of mechanical shear in promoting protein alignment and network consolidation. However, this effect was not statistically significant (*p* > 0.05) at higher barrel temperatures (samples 6 and 11) or higher moisture levels (samples 12 and 15), indicating that thermal softening and increased hydration can offset shear-induced strengthening. Barrel temperature also influenced textural behavior in a condition-dependent manner. Under low moisture level and low screw speed (samples 2 and 3), increasing barrel temperature led to higher TPA values, likely due to enhanced protein denaturation and intermolecular interactions. Under other processing conditions, however, no consistent pattern was observed.

Cutting strength exhibited a generally positive correlation with barrel temperature, suggesting that thermal energy contributed to the formation of a further resistant protein network capable of withstanding compressive and shear forces. Overall, these results demonstrate that the textural properties of the meat alternatives are governed by complex interactions among rotational speed of the screw, moisture level, and processing temperature. The balance between hydration, mechanical shear, and thermal input determines the extent of protein network development, ultimately controlling both elastic and fracture-related textural attributes.

### 2.5. Optimization of Process Variables

**Figure 2:**
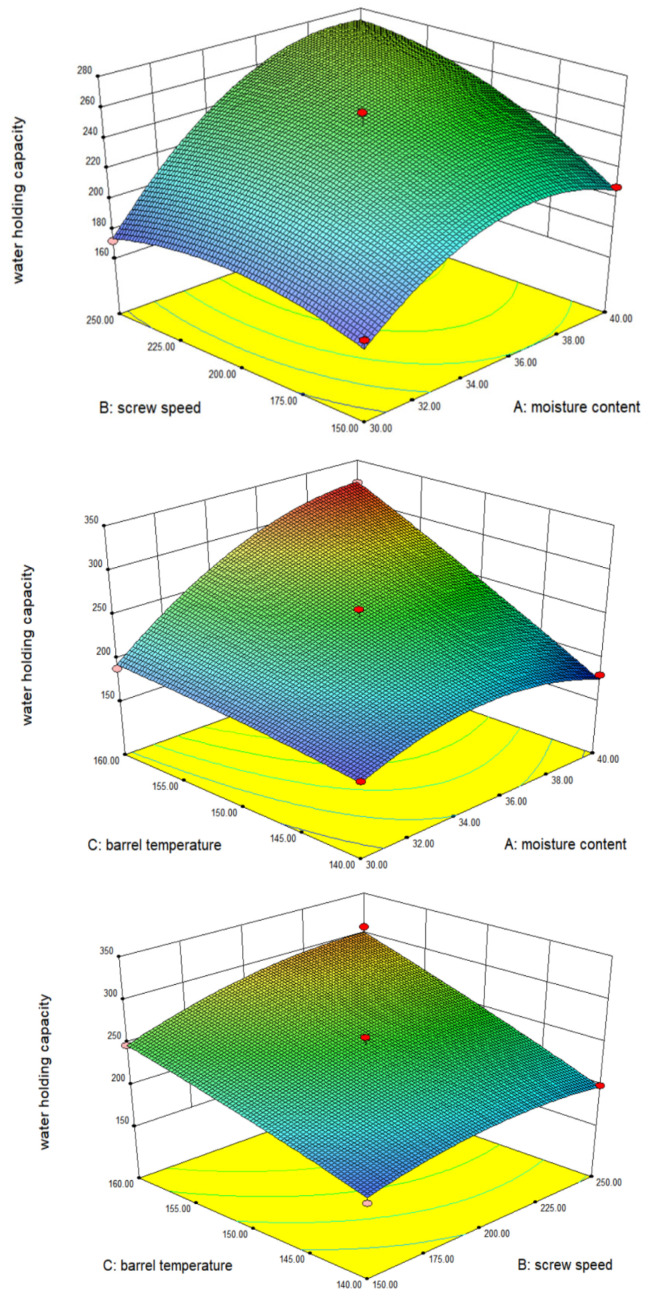
Three-dimensional response surface plots indicating the effects of moisture level, barrel temperature, and screw speed on the water-holding capacity of meat alternatives.

Water-holding capacity (WHC) is a key functional marker for evaluating the quality and structural performance of meat analogs, as it reflects the ability of the protein network to immobilize water within the matrix. The influences of extrusion processing parameters, rotational speed of the screw, moisture level, and processing temperature on WHC of meat analogs were quantified using a quadratic polynomial model, as summarized in Table 3. Three-dimensional response surface plots illustrating the combined impact of processing parameters on WHC are presented in Figure 2. The fitted model adequately described WHC behavior, showing high significance (*p* < 0.01) and no evidence of lack of fit (*p* > 0.05). Moisture content, rotational speed of the screw, and barrel temperature all exerted significant effects on WHC (*p* < 0.01). In addition, the relations between moisture content and barrel temperature, as well as the quadratic term of moisture content, showed highly significant contributions, highlighting the dominant role of hydration in governing water retention behavior.

Among the investigated factors, moisture content exhibited the strongest influence on WHC, followed by rotational speed of the screw and processing temperature. This hierarchy reflects the critical role of water availability in facilitating protein hydration, gel network formation, and pore development during extrusion, which collectively determine the capacity of the extrudate to retain water. Barrel temperature contributed by modulating protein denaturation and network consolidation, while screw speed played a secondary role by influencing mechanical shear and expansion behavior. Based on the response surface model, the model predicted maximum WHC at a moisture level of 39.45%, a processing temperature of 159.41 °C, and a screw rotational speed of 215.74 rpm. For practical operation and experimental reproducibility, these values were rounded to 39% moisture content, 159 °C barrel temperature, and 216 rpm screw speed for validation experiments. These optimized conditions correspond to a processing window that promotes the development of a stable, gel-like protein network with enhanced water retention capacity in IMBP-based LMMAs.

## 3. Conclusions

This study systematically explored the effects of extrusion process parameters on the structural and functional properties of isolated mung bean protein-based low-moisture meat analogs (IMBP-based LMMAs), with a particular focus on extrusion-induced gel network formation. Rotational speed of the screw, moisture level, and processing temperature were identified as critical factors governing pore development and water-holding capacity, highlighting the critical role of hydration and thermomechanical input in shaping the internal structure of the extrudates. Higher moisture level promoted the formation of larger and more abundant pores, which enhanced water immobilization within the protein matrix and resulted in improved water-holding capacity. In contrast, the integrity index remained relatively insensitive to variations in screw speed and barrel temperature under low-moisture extrusion conditions, indicating that the macroscopic cohesion of the protein network was maintained across the investigated processing window. Nitrogen solubility index (NSI) responded more sensitively to changes in moisture content and screw speed, reflecting extrusion-induced protein denaturation and aggregation that modulated the balance between soluble and insoluble protein fractions. Response surface analysis enabled the identification of an optimal processing window for maximizing water-holding capacity, corresponding to a screw speed of 216 rpm, a moisture content of 39%, and a barrel temperature of 159 °C. These conditions favor the development of a consolidated, gel-like protein network that provides both structural stability and functional performance. Overall, the findings demonstrate that controlled extrusion-induced gelation of mung bean protein can be effectively tuned through process variable optimization to achieve desirable structure–property relationships in low-moisture meat analogs. This work provides mechanistic insight into protein network development during low-moisture extrusion and offers practical guidance to produce high-quality, plant-based meat alternatives aligned with the increasing demand for sustainable foods.

## 4. Materials and Methods

### 4.1. Materials

Isolated mung bean protein (IMBP; Harbin Hada Starch Co., Ltd., Heilongjiang, China), wheat gluten (WG; Roquette Frères, Lestrem, France), and corn starch (CS; Samyang Ltd., Ulsan, Republic of Korea) served as the main raw materials for the preparation of low-moisture meat analogs. The formulation ratio of IMBP, WG, and CS was fixed at 50:40:10 (*w*/*w*/*w*).

### 4.2. Extrusion Process

**Figure 3:**
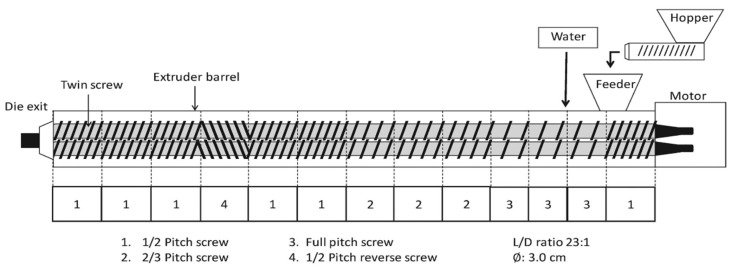
Extruder setup for low-moisture extrusion.

Low-moisture meat analogs (LMMAs) were produced via a co-rotating twin-screw extruder (Incheon Machinery Co., Ltd., Incheon, Republic of Korea). The extruder was equipped with screws having a diameter of 30 mm and a total length of 690 mm. A schematic representation of the extrusion system is shown in Figure 3. Low-moisture extrusion cooking (LMEC) was conducted using a short-slit die with dimensions of 1.0 × 0.45 × 8.0 cm (width × height × length). The extrusion conditions were systematically adjusted in accordance with the experimental design, and the corresponding parameter settings are presented in Table 4. The feeding rate was maintained constant at 100 g/min. Following extrusion, the products were cut into uniform lengths and dried in a hot-air dryer at 50 °C for 7 h. Dried samples were either retained in their original form or ground using a stainless-steel mixer, depending on the subsequent analyses. Both intact and powdered samples were sealed in plastic bags and stored at room temperature.

### 4.3. Water-Holding Capacity (WHC)

The water-holding capacity (WHC) of low-moisture meat analogs (LMMAs) was determined based on an established method with slight modifications [17]. Extruded samples were cut into cubes with a side length of 1 cm. Samples corresponding to 5 g were immersed in 100 mL of water and thermally treated in a water bath at 90 °C for 40 min. And then, excess surface water was removed by draining the samples on a 20-mesh sieve for 15 min. WHC was calculated using Equation (1). Each analysis was repeated three times, and the results are reported as mean ± SD.

WHC (%) = (M_2_ − M_1_)/M_1_ × 100% (1)

where M_1_ represents the weight of the dry sample and M_2_ denotes the weight after rehydration.

### 4.4. Integrity Index

The integrity index of meat alternatives was measured using a modified procedure based on previously reported methods [17,18]. Extruded samples were cut into cubes of approximately 1 cm × 1 cm. Portions corresponding to a dry weight (W_a_) of 5.0 g were transferred into conical flasks containing 100 mL of distilled water. After heating at 90 °C for 30 min in a water bath, the samples were subjected to autoclave treatment (PAC-60, Lab House Co., Ltd., Seoul, Republic of Korea) at 121 °C for 15 min. Following autoclave treatment, the extrudates were placed on a mesh sieve (W_b_), rinsed under tap water for 1 min, and subjected to high-speed homogenization at 17,530× *g* for 1 min (T10 Basic, IKA Co., Ltd., Seoul, Republic of Korea). The homogenized samples were rinsed again on the same sieve with water for 1 min. Subsequently, sieve containing the retained residues was dried at 105 °C until a constant weight (W_c_) was achieved. The integrity index was calculated using Equation (2). All measurements were performed in triplicate.

Integrity index (%) = (W_c_ − W_b_)/W_a_ × 100% (2)

### 4.5. Nitrogen Solubility Index (NSI)

The nitrogen solubility index (NSI) of low-moisture meat analogs (LMMAs) was determined following a previously reported protocol with slight modifications [18]. Soluble nitrogen was extracted by mixing 0.2 g of the powdered sample with 10 mL of 0.5% (*w*/*v*) KOH, followed by shaking for 30 min in an incubator. For total nitrogen determination, 0.2 g of powdered sample was mixed with 5 mL of 6 N HCl and hydrolyzed at 100 °C for 24 h. After hydrolysis, samples were brought to volume with distilled water (10 mL) and subsequently treated with centrifugation at 3000 rpm for 30 min. Supernatants were collected. An aliquot of 0.05 mL of each supernatant was reacted with 1.67 mL of ninhydrin reagent and held at 100 °C for 10 min. A UV–visible spectrophotometer measured absorbance at 575 nm. NSI was calculated according to Equation (3). All measurements were performed in triplicate.

NSI (%) = (Soluble nitrogen/Total nitrogen) × 100%(3)

### 4.6. Texture Profile Analysis and Cutting Strength

Texture profile analysis (TPA) of low-moisture meat analogs (LMMAs) was performed by a texture analyzer (Compac-100, Sun Science Co., Tokyo, Japan). Extruded samples were cut into cubes with dimensions of approximately 1 cm × 1 cm × 1 cm, rehydrated in water at 90 °C for 30 min. The samples were allowed to drain in a mesh sieve for 15 min. Rehydrated samples’ dimensions (height × length × width) were measured using a digital vernier caliper (CD-15CPX, Mitutoyo Co., Ltd., Kawasaki, Japan). TPA measurements were conducted using a cylindrical compression probe with a diameter of 25 mm. Samples were subjected to a double-compression test under the following conditions: maximum load of 10 kg, crosshead speed of 100 mm min^−1^, and a compression distance of 15 mm between the two compression plates. Springiness and cohesiveness were calculated according to Equations (4) and (5), and chewiness was calculated by multiplying peak stress, springiness, and cohesiveness as described previously [28].

Cutting strength was measured by a probe (7.5 mm × 38.3 mm) under a 2 kg maximum-load setting. Measurements were conducted in both the vertical and parallel directions relative to the extrusion flow, and cutting strength values were calculated using Equation (6).

Springiness (%) = D_2_/D_1_ × 100% (4)

in which D_1_ denotes the position of the initial maximum stress, while D_2_ denotes that of the subsequent maximum stress.

Cohesiveness (%) = A_2_/A_1_ × 100% (5)

in which A_1_ denotes the work area obtained during the initial compression cycle, while A_2_ denotes that measured during the subsequent compression cycle.

Cutting strength (g/cm^2^) = Peak stress/Cross-sectional area (6)

### 4.7. Experimental Design and Data Analysis

A Box–Behnken scheme within the response surface methodology framework was employed to define the experimental plan. The experimental design, statistical evaluation, and regression modeling were carried out using Design-Expert (version 8.0.6, Stat-Ease Inc., Minneapolis, MN, USA). The study considered three independent factors with three levels assigned to each factor. A total of 15 experimental runs, including three center-point replications, were generated according to the Box–Behnken configuration and conducted in a randomized order to minimize systematic bias. Water-holding capacity of IMBP-based low-moisture meat analogs (LMMAs) was selected as the response variable (Y). Analysis of variance (ANOVA) was performed to evaluate the significance of linear, quadratic, and interaction effects of the independent variables. The adequacy of the regression model and the significance of each term were assessed using F-values at probability levels of *p* < 0.05 and *p* < 0.01. Regression coefficients obtained from the fitted model were used to generate contour and three-dimensional response surface plots.

Further statistical evaluation was carried out via IBM SPSS Statistics (v22.0; IBM, Armonk, NY, USA). Mean comparisons among treatments were performed using Duncan’s multiple range test, and differences were considered statistically significant at *p* < 0.05.

## Figures and Tables

**Table 1:** Influences of extrusion process parameters on WHC, integrity index, and NSI of meat alternatives.

Run Order	X_1_^(1)^	X_2_	X_3_	WHC (%) ^(2)^	Integrity Index (%)	NSI (%)
	MC (%)	SS (rpm)	BT (°C)			
1	30	150	150	173.58 ± 21.26 ^f(3)^	71.73 ± 1.38 ^cde^	29.58 ± 0.52 ^c^
2	30	200	140	171.39 ± 13.58 ^f^	69.77 ± 0.96 ^e^	30.91 ± 2.41 ^bc^
3	30	200	160	188.78 ± 17.41 ^def^	78.36 ± 2.41 ^a^	31.12 ± 0.81 ^b^
4	30	250	150	171.95 ± 5.06 ^f^	79.20 ± 1.39 ^a^	26.24 ± 2.56 ^d^
5	35	150	140	172.23 ± 10.45 ^f^	72.49 ± 4.15 ^cde^	25.19 ± 0.98 ^d^
6	35	150	160	248.30 ± 14.83 ^bc^	75.03 ± 0.34 ^bc^	25.11 ± 0.91 ^d^
7	35	200	150	257.01 ± 7.89 ^bc^	73.22 ± 0.89 ^cde^	32.94 ± 2.05 ^abc^
8	35	200	150	248.00 ± 8.03 ^bc^	74.39 ± 1.14 ^bcd^	33.20 ± 2.54 ^ab^
9	35	200	150	240.63 ± 5.26 ^c^	71.52 ± 0.84 ^cde^	34.83 ± 0.27 ^a^
10	35	250	140	198.33 ± 4.64 ^de^	71.00 ± 0.32 ^de^	24.49 ± 1.26 ^d^
11	35	250	160	308.53 ± 20.23 ^a^	72.29 ± 0.97 ^cde^	32.24 ± 2.81 ^abc^
12	40	150	150	207.60 ± 13.64 ^d^	72.84 ± 1.60 ^cde^	31.99 ± 1.14 ^abc^
13	40	200	140	179.70 ± 681 ^ef^	69.72 ± 3.58 ^e^	24.43 ± 3.43 ^d^
14	40	200	160	323.22 ± 7.41 ^a^	72.22 ± 1.03 ^cde^	35.35 ± 1.87 ^a^
15	40	250	150	266.62 ± 8.71 ^b^	77.32 ± 1.22 ^ab^	30.69 ± 0.98 ^bc^

^(1)^ X1, moisture content; X2, screw speed; X3, barrel temperature. ^(2)^ WHC, water-holding capacity; NSI, nitrogen solubility index. ^(3)^ Values sharing different superscript letters in each column are significantly different (*p* < 0.05) according to Duncan’s multiple range test.

**Table 2:** Influences of extrusion process parameters on TPA and cutting strength of meat alternatives.

Run Order	X_1_^(1)^	X_2_	X_3_	TPA ^(2)^			Cutting Strength (g/cm^2^)	
	MC (%)	SS (rpm)	BT (°C)	SPR (%)	COH (%)	CHW (g)	VCS	PCS
1	30	150	150	77.45 ± 3.52 ^e(3)^	49.55 ± 8.99 ^f^	1056.29 ± 153.63 ^de^	501.08 ± 33.62 ^e^	375.31 ± 69.23 ^e^
2	30	200	140	79.88 ± 1.90 ^e^	53.73 ± 5.40 ^f^	822.83 ± 156.77 ^g^	353.87 ± 34.67 ^g^	287.44 ± 46.23 ^f^
3	30	200	160	84.20 ± 5.31 ^d^	69.63 ± 5.59 ^cd^	1088.17 ± 166.09 ^cde^	529.88 ± 53.30 ^e^	514.15 ± 43.69 ^bcd^
4	30	250	150	88.99 ± 2.64 ^abc^	78.30 ± 8.47 ^ab^	1604.65 ± 204.47 ^a^	662.45 ± 54.30 ^b^	555.68 ± 40.54 ^b^
5	35	150	140	83.78 ± 1.69 ^d^	65.62 ± 2.95 ^de^	1029.14 ± 138.43 ^def^	487.65 ± 42.49 ^e^	577.87 ± 49.83 ^b^
6	35	150	160	84.16 ± 2.84 ^d^	72.21 ± 3.41 ^bcd^	427.89 ± 127.70 ^i^	584.83 ± 31.40 ^d^	477.40 ± 39.82 ^cd^
7	35	200	150	88.20 ± 2.26 ^abc^	71.93 ± 2.11 ^bcd^	879.06 ± 98.13 ^fg^	597.18 ± 28.55 ^cd^	471.66 ± 53.05 ^d^
8	35	200	150	89.38 ± 1.80 ^abc^	75.06 ± 3.61 ^abc^	573.51 ± 69.13 ^hi^	647.23 ± 27.04 ^bc^	565.46 ± 61.36 ^b^
9	35	200	150	86.73 ± 4.89 ^bcd^	76.19 ± 5.64 ^abc^	627.73 ± 49.14 ^h^	580.57 ± 24.41 ^d^	589.64 ± 36.46 ^b^
10	35	250	140	89.03 ± 1.86 ^abc^	73.88 ± 3.03 ^abc^	1272.35 ± 161.04 ^b^	597.48 ± 15.39 ^cd^	549.22 ± 23.45 ^bc^
11	35	250	160	86.46 ± 2.51 ^cd^	78.81 ± 1.03 ^ab^	501.31 ± 181.94 ^hi^	887.30 ± 32.63 ^a^	718.60 ± 36.84 ^a^
12	40	150	150	90.48 ± 1.56 ^ab^	71.01 ± 4.31 ^cd^	1166.62 ± 150.13 ^bcd^	604.76 ± 66.47 ^cd^	568.64 ± 46.99 ^b^
13	40	200	140	83.55 ± 3.33 ^d^	60.97 ± 9.5 2 ^e^	1256.53 ± 129.71 ^bc^	414.19 ± 33.19 ^f^	532.18 ± 130.59 ^bcd^
14	40	200	160	88.81 ± 1.67 ^abc^	72.77 ± 2.74 ^bc^	432.10 ± 74.52 ^i^	626.83 ± 32.45 ^bcd^	518.74 ± 58.50 ^bcd^
15	40	250	150	90.99 ± 1.25 ^a^	79.79 ± 1.26 ^a^	943.43 ± 132.41 ^efg^	631.89 ± 46.11 ^bcd^	587.02 ± 57.41 ^b^

^(1)^ X1, moisture content; X2, screw speed; X3, barrel temperature. ^(2)^ TPA, texture profile analysis; SPR, springiness; COH, cohesiveness; CHW, chewiness; VCS, vertical cutting strength; PCS, parallel cutting strength. ^(3)^ Values sharing different superscript letters in each column are significantly different (*p* < 0.05) according to Duncan’s multiple range test.

**Table 3:** ANOVA results for regression models describing the WHC of meat analogs.

Source	WHC ^(1)^	
	*p*-Value	Significance
Model	0.0002	** ^(2)^
X_1_-moisture content	<0.0001	**
X_2_-screw speed	0.0017	**
X_3_-barrel temperature	<0.0001	**
X_1_ X_2_	0.0147	*
X_1_ X_3_	0.0006	**
X_2×3_	0.0949	
X_1_^2^	0.0010	**
X_2_^2^	0.0244	*
X_3_^2^	0.5272	
Lack of Fit	0.5247	
R^2^	0.9905	
Adj R^2^	0.9733	
C.V. %	3.71	

^(1)^ WHC, water-holding capacity. ^(2)^ *, significant at 0.05 level; **, significant at 0.01 level.

**Table 4:** A central composite approach for optimizing extrusion processing conditions.

Run Order	X_1_	X_2_	X_3_
	Moisture Content (%)	Screw Speed (rpm)	Barrel Temperature (°C)
1	30	150	150
2	30	200	140
3	30	200	160
4	30	250	150
5	35	150	140
6	35	150	160
7	35	200	150
8	35	200	150
9	35	200	150
10	35	250	140
11	35	250	160
12	40	150	150
13	40	200	140
14	40	200	160
15	40	250	150
16	30	150	150

## Data Availability

The original contributions presented in this study are included in the article. Further inquiries can be directed to the corresponding author.
